# Effects of NSAIDs on Differentiation and Function of Human and Murine Osteoclasts – Crucial ‘Human Osteoclastology’

**DOI:** 10.3390/ph3051394

**Published:** 2010-05-11

**Authors:** Shigeru Kotake, Toru Yago, Manabu Kawamoto, Yuki Nanke

**Affiliations:** Institute of Rheumatology, Tokyo Women's Medical University, 10-22 Kawada-cho, Shinjuku-ku, Tokyo 162-0054, Japan; E-Mails: toruyago@yahoo.co.jp (T.Y.); kawamoto@ior.twmu.ac.jp (M.K.); ynn@ior.twmu.ac.jp (Y.N.)

**Keywords:** non-steroidal anti-inflammatory drugs (NSAIDs), osteoclast, osteo-clastogenesis, osteoclastology, prostaglandin E2 (PGE2), interleukin-17 (IL-17)

## Abstract

Osteoclasts play a critical role in both normal bone metabolism and bone resorption in the joints of patients with rheumatoid arthritis. It has been reported that non-steroidal anti-inflammatory drugs (NSAIDs) inhibit murine osteoclastogenesis *in vitro* and murine arthritis models *in vivo*, but not the destruction of joints of patients with rheumatoid arthritis. In the current review article, we review the recent findings in the effect of NSAIDs on the formation and function of human and murine osteoclasts both *in vitro* and *in vivo*, underlining the importance of studies using human osteoclasts. Since 2009, we have suggested a novel term ‘human osteoclastology’.

## 1. Introduction

Many studies have investigated the hypothesis that activated T cells directly or indirectly modulate the formation and function of osteoclasts in bone resorption associated with rheumatoid arthritis (RA) since the receptor activator of NF-κB ligand (RANKL) was cloned as a factor inducing osteoclastogenesis in 1997–8. These findings of RANKL were the breakthrough in the study of osteoclastogenesis. We and others have reported that activated T cells expressing RANKL induce osteoclastogenesis [1,2,3]. In addition, in 1999, we demonstrated that interleukin-17 (IL-17), which was first cloned in 1995, potently induces murine osteoclastogenesis via the expression of RANKL on murine osteoblasts, and that IL-17 is present in synovial fluid and tissues from patients with RA [4]. IL-17 induces RANKL expression on murine osteoblasts via the expression of prostaglandin E2 (PGE2) [4]. IL-1β or tumor necrosis factor α (TNF-α) also induces RANKL on osteoblasts via the expression of PGE2. Thus, PGE2 plays a pivotal role in inducing osteoclastogenesis in lesions with inflammation. 

The functions of various human factors, including cytokines or prostaglandins, were recently reported to be different from those of mice [5]. Surprisingly, it has been reported that human PGE2 potently inhibits human osteoclastogenesis from monocytes alone induced by RANKL [6]. Thus, it is very important to underscore the differences between humans and mice when we evaluate the findings of experiments using mice.

In the current article, we first review the mechanism of osteoclastogenesis and then the effects of non-steroidal anti-inflammatory drugs (NSAIDs) on the differentiation and activity of human and murine osteoclasts both *in vitro* and *in vivo*. Systemic effects of NSAIDs on bone metabolism reflect the balance of bone resorption by osteoclasts and bone formation by osteoblasts. Thus, we also review the effects of NSAID on osteoblasts. According to recent findings, it might be recommended that NSAIDs should be more carefully used in patients with RA, osteoporosis, and bone fracture.

## 2. Osteoclasts Are Formed From Monocytes Stimulated by RANKL

**Figure 1:**
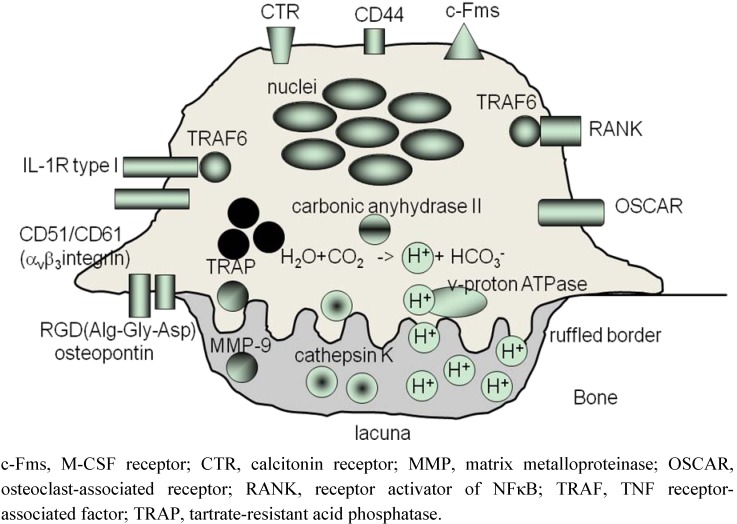
Schematic structure of osteoclasts.

Osteoclasts are unique multinucleated cells whose specialized function is to resorb calcified tissues (Figure 1) [7]. On the surface of bone, osteoclasts develop a specialized adhesion structure, the ‘podosome’, which subsequently undergoes reorganization into sealing zones [8]. These ring-like adhesion structures, *i.e.*, actin rings, seal osteoclasts to the surface of bone. In the sealed resorption lacuna, localized acidification is driven by carbonic anhydrase II and vacuolar H(+)-ATPase in osteoclasts; carbonic anhydrase II produces protons and vacuolar H(+)-ATPase transfers them into the lacuna. In acidified lacuna, cathepsin-K and matrix metalloproteinase-9 (MMP-9) are released from osteoclasts to degrade calcified tissues [9]. 

The cooperation of osteoclasts and osteoblasts is critical to maintain skeletal integrity in normal bones. After bone resorption by osteoclasts on normal bone tissues, osteoblasts subsequently rebuild bone in the lacunae resorbed by osteoclasts; this mechanism is called ‘bone remodeling’. When the activity or number of osteoclasts is elevated compared with osteoblasts, the bone becomes fragile, that is, ‘osteoporotic’. In addition, bone remodeling is disrupted in all bone diseases associated with changes in bone mass. Thus, bone remodeling is essential to retain both the structure and strength of normal bone.

Osteoclasts also play an important role in the pathogenesis of rheumatoid arthritis (RA). Since 1984, it has been reported that in bone destruction of rheumatoid arthritis (RA), many activated osteoclasts are detected on the surface of eroded bone in the interface with synovial tissues [10]. In addition, we have demonstrated that osteoclasts are detected in synovial tissues as well as eroded bone from patients with RA [11]. In addition, we have reported that the number of precursor cells of osteoclasts increases in bone marrow adjacent to joints with arthritis [12]. Moreover, the amount of cytokine that induces osteoclastogenesis, such as IL-1, TNF-α and IL-6, is elevated in synovial tissues of patients with RA, while the amount of cytokine that inhibit osteoclastogenesis, such as IL-4 and IL-10, is decreased [12,13,14,15]. Thus, patients with RA are likely to suffer from joint destruction as well as systemic osteoporosis, in which the number of osteoclasts increases, suggesting that osteoclasts play a critical role in the pathogenesis of RA. 

The origin of osteoclasts was unclear until the late 1980s. In 1988, Takahashi *et al.* established a co-culture system using mouse spleen cells and osteoblasts to induce osteoclastogenesis *in vitro*, demonstrating that the origin of osteoclasts is hematopoietic cells and that osteoblastic cells are required for the differentiation of osteoclast progenitors in splenic tissues into multinucleated osteoclasts [16]. The precursor of osteoclasts was then revealed to be colony-forming unit–macrophage (CFU–M) or CFU–granulocyte/macrophage (CFU–GM) in bone marrow or spleen in mice. In 1990, Udagawa *et al.* demonstrated that osteoclasts are formed from murine macrophages [17]. From these findings, Suda *et al.* hypothesized that bone marrow hemopoietic cells differentiate into osteoclasts through the stimulation of ‘osteoclast-differentiation factor (ODF)’ expressed on osteoblasts [18].

**Figure 2:**
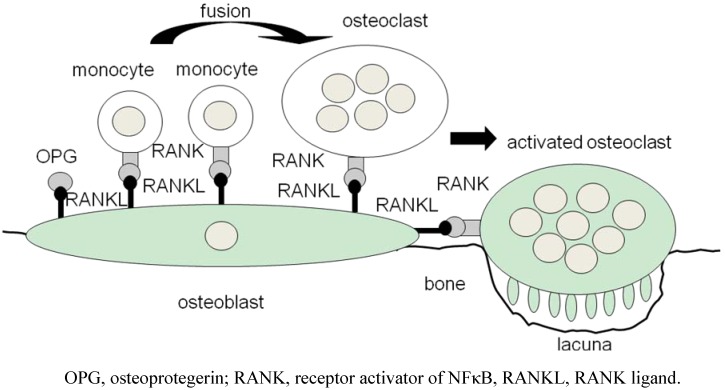
Differentiation and activation of osteoclasts. A RANK-RANKL system induces both osteoclastogenesis from monocytes and the activation of mature osteoclasts.

Finally, ODF, now termed RANKL, which induces osteoclastogenesis from monocytes or macrophages, was independently cloned by three groups in 1997 (Figure 1) [19]. RANKL is a member of the TNF superfamily of cytokines. The protein constructs a trimeric complex to bind its receptor, receptor activator NF-κB (RANK) [20]. A decoy receptor is also cloned, which is designated as ‘osteoprotegerin (OPG)’ [19] (Figure 2). In 2000–2001, we and other groups showed that T cells expressing RANKL induce osteoclastogenesis [1,2,3]; in particular, we demonstrated osteo-clastogenesis using human cells [3], whereas others used murine cells [1,2]. In addition, in 2009, we reported that, in human osteoclastogenesis induced by RANKL, T-cell leukemia translocation-associated gene (TCTA) protein is required for cellular fusion [21].

## 3. ‘Human Osteoclastology’

In basic science, murine cells are usually used, because these cells and experimental tools can be easily obtained. In addition, it is possible to create transgenic and knockout murine models. On the other hand, there are many disadvantages in studies using human cells, e.g., they are difficult to obtain. In addition, it is impossible to perform an *in vivo* study.

However, to investigate the pathogenesis of RA, it is critical to investigate osteoclastogenesis using human cells. In bone cell biology, some cells or cytokines show different functions between humans and mice. For example, the mouse CD4+CD25+ regulatory subset can be isolated from all CD25+ T cells regardless of their level of CD25 expression; however, when similar criteria are followed to isolate these cells from human blood, CD25+ cells (high and low together) do not exhibit an aneroid phenotype or significant suppressive function. In 2001, Baecher-Allan *et al.* demonstrated that, in humans, CD4+CD25hi exhibit all the properties of regulatory T cells [22]. We also measured the percentages of CD4+CD25hi T cells as regulatory T cells in patients with Behcet’s disease [23]. Thus, in the study of human diseases, it is essential to investigate human osteoclastogenesis using human cells; differences in the species used in studies are critical to discuss the function of cytokines [5]. In 2009, we therefore suggested that the term ‘human osteoclastology’ be used to describe studies on human osteoclastogenesis [5]. 

## 4. Roles of PGE2 in Osteoclastogenesis

### 4.1. Development of culture systems to form osteoclasts in vitro

Culture systems were developed to form osteoclasts *in vitro* in 1981–1988. In 1981, Testa *et al.* first succeeded in forming osteoclast-like multinucleated cells from feline marrow cells in long-term Dexter cultures [24]. In 1984, using this feline marrow culture system, Ibbotson *et al.* showed that the formation of osteoclast-like cells is greatly stimulated by osteotropic hormones, such as 1,25(OH)2D3, PTH, and prostaglandin E2 (PGE2) [25]. In 1987, MacDonald *et al.* reported the formation of multinucleated cells that respond to osteotropic hormones in long-term human bone marrow cultures [26]. In 1988, Takahashi *et al.* and in 1989, Hattersley *et al.* used marrow cells of mice to examine osteoclast-like cell formation from their progenitor cells [27,28]. Moreover, in 1988, Takahashi *et al.* established an innovative co-culture system using mouse spleen cells and osteoblasts to induce osteoclastogenesis *in vitro* [16]. Thus, since 1981, studies using osteoclastogenesis *in vitro* have been developed, and PGE2 was shown to up-regulate murine osteoclastogenesis using the marrow culture system *in vitro* as follows.

### 4.2. Role of PGE2 in murine osteoclastogenesis

PGE2 is a major product among several prostaglandins produced in bone. PGE2 production by osteoblasts is regulated by several cytokines, including IL-1. Sato *et al.* reported that rhIL-1αstimulates bone resorption, which is partially inhibited by indomethacin, and that PGE2 produced in the bone is at least in part involved in osteoclast bone resorption using fetal mouse bones [29]. Thus, PGE2 has been shown to induce bone resorption in experiments using mouse bones.

PGE2 induces mouse osteoclastogenesis from the mouse macrophage-like cell line, RAW264.7 cells, regulating the OPG/RANKL/RANK system in the interaction between osteoblasts and osteoclasts. Liu *et al.* reported that cyclooxygenase (COX)-2 and PGE2 stimulate osteoclastogenesis through the stimulation of RANKL production and inhibition of OPG secretion by osteoblasts, and up-regulation of RANK expression in osteoclasts using murine cells [30]. Interestingly, Liu *et al.* also showed that PGE2 simulates the formation of TRAP+ multinucleated cells from RAW264.7 cells alone, indicating the direct effect of PGE2 on osteoclast progenitors. In addition, they demonstrated that cross talk between endogenous IL-6 and PGE2 signaling systems results in the enhancement of osteoclastogenesis through effects on the OPG/RANKL/RANK system.

PGE2 directly promotes mouse osteoclastogenesis induced by RANKL. Wani *et al.* demonstrated that PGE2 cooperates with RANKL in mouse osteoclast formation from hematopoietic precursors in the absence of osteoblasts, inducing synergistic activation of differentiation, cell spreading, and fusion [31]. In addition, in 2005, Kobayashi *et al.* reported that the effect of PGE2 on RANKL-induced osteoclast differentiation in mouse bone marrow macrophage cultures is mediated through EP2 and EP4 [32]. They also showed that TGF-beta-activated kinase 1 (TAK1) acted as an adapter molecule, linking PKA-induced signals and RANKL-induced signals in mouse osteoclast precursors. In 2005, Han *et al.* reported that RANKL selectively induces COX-2 expression via Rac1, which in turn results in the production of PGE2 in mouse osteoclast precursors, RAW264.7 cells [33]. Thus, in addition to the indirect effect of PGE2 on osteoblastic cells, PGE2 play a direct role in mouse osteoclastogenesis. In other words, PGE2 stimulates osteoclastic bone resorption in mice through two pathways: PGE2 induces (1) RANKL expression by osteoblasts, and (2) direct enhancement of RANKL-induced osteoclastogenesis from the precursors.However, there is no convincing evidence that, in the absence of RANKL, PGE2 acts directly on osteoclast precursors and stimulate osteoclasts differentiation.

The biphasic effect of PGE2 has been reported in osteoclast formation in mouse spleen cell cultures induced by RANKL. Ono *et al.* reported that PGE2 has an initial inhibitory effect on osteoclast formation in mouse spleen cell cultures, possibly mediated by both EP2 and EP3 receptors, and a later stimulatory effect, mediated by the EP2 receptor, possibly acting on T-cells [34]. Thus, these findings suggest that the effects of PGE2 on mouse osteoclastogenesis depend on the time when the concentration of PGE2 increases. 

### 4.3. Role of PGE2 in human osteoclastogenesis

**Figure 3:**
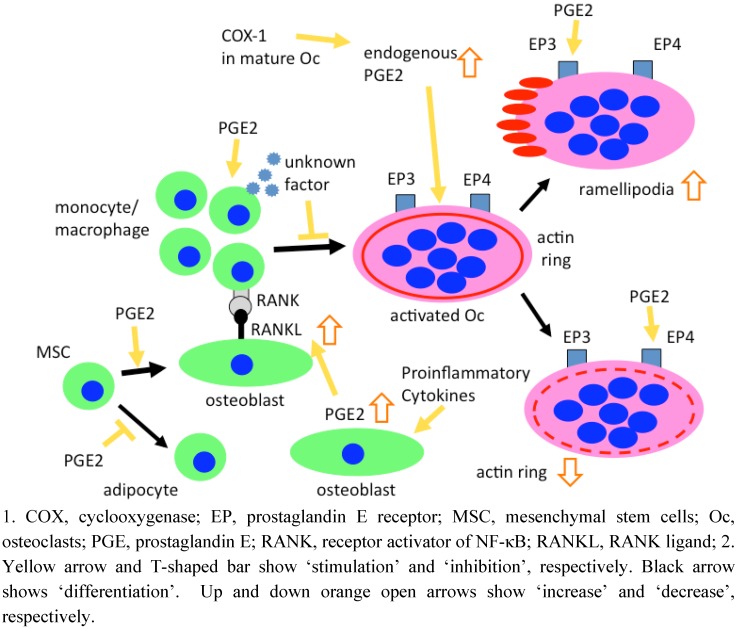
Effects of PGE2 on formation and function of human osteoclasts, and human osteoblastogenesis.

The roles of PGE2 in human osteoclasts have been reported both *in vivo* and *in vitro*. Children with hyperprostaglandin E syndrome, a neonatal variant of Bartter syndrome with enhanced renal and systemic formation of PGE2, have hypercalciuria, nephrocalcinosis, and osteopenia [35]. On the other hand, the effects of PGE2 on human osteoclastogenesis *in vitro* were also investigated using co-culture methods. In 2000, Naele *et al.* reported that PGE2 stimulates osteoclastogenesis using co-culture with human monocytes and human bone-derived stromal cells [36]. Thus, similar to the effect of PGE2 on murine osteoclastogenesis, PGE2 has been shown to stimulate the formation and function of human osteoclasts indirectly via osteoblasts. PGE2 was also shown to promote an increase in the ratio of RANKL to OPG in human osteoblastic stromal cells [37,38] (Figure 3). In addition, in 1988, Lader and Flanagan reported that PGE2 increase osteoclastogenesis from human bone marrow cell cultures [39]. Thus, in human co-cultures with osteoblastic cells and osteoclast precursor cells or human bone marrow cell cultures, PGE2 also induces osteoclastogenesis indirectly via RANKL expression on osteoblastic cells.

Only two groups have reported the effects of PGE2 on human osteoclastogenesis from monocytes alone stimulated by RANKL in the absence of osteoblasts, contrary to many reports on the stimulatory effects of PGE2 on murine cells. In 1999, Itonaga *et al.* reported that PGE2 inhibits osteoclast formation induced by RANKL in human peripheral blood mononuclear cell cultures [40]. In 2005, Take *et al.* demonstrated that, unlike mouse macrophage cultures, PGE2 strongly inhibits RANKL-induced osteoclast formation in human CD14+ cell cultures [6] (Figure 3). In addition, they showed that human osteoclast progenitors produce a soluble unidentified factor(s) in response to PGE2 that strongly inhibits RANKL-induced osteoclast formation not only in human CD14+ cell cultures but also in mouse macrophage cultures. They tried to identify the soluble factors, and concluded that CD14+ cells produce an inhibitor(s) that does not correspond to known inhibitory factors, such as GM-CSF, IFN-γ, and IL-4. Thus, these reports demonstrated the possibility that PGE2 differently plays a direct role in osteoclastogenesis from monocytes alone in the absence of osteoblastic cells between humans and mice.

## 5. IL-17 Induces Osteoclastogenesis via the Expression of PGE2 in Osteoblastic cells

**Figure 4:**
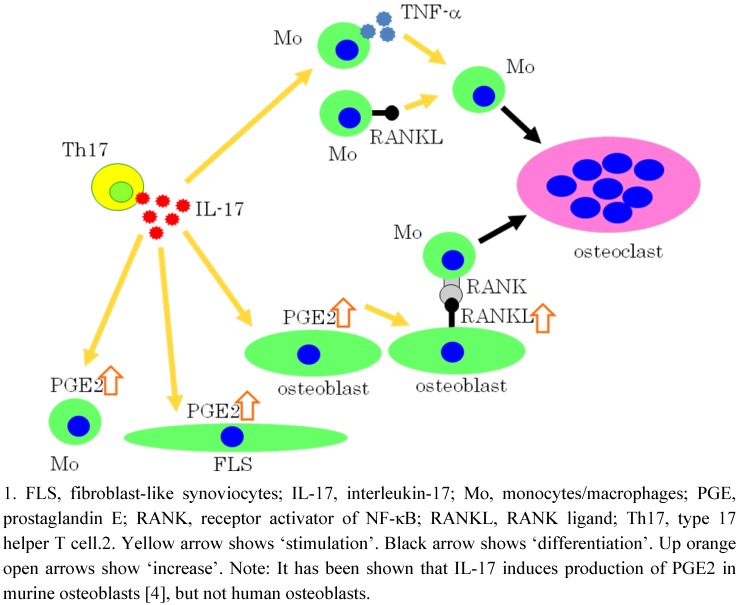
Direct and indirect effects of IL-17 on osteoclastogenesis.

In 1999, we first demonstrated that IL-17 potently induces osteoclastogenesis from monocytes through the expression of RANKL on osteoblasts using mouse cells [4] (Figure 4). The expression of RANKL is induced by PGE2 in osteoblasts induced by IL-17, which is inhibited by NS398, a selective COX-2 inhibitor. Thus, IL-17 indirectly induces osteoclastogenesis via osteoblasts by this mechanism. In 2008, Kwan Tat *et al.* reported, using human osteoblasts, that membranous RANKL levels are increased approximately 2.5-fold compared with controls when incubated with PGE2 or IL-17, although this reaches statistical significance in PGE2, but not in IL-17 [41] (Figure 4). In addition, in 2004, Stamp *et al.* reported that IL-17 produced by activated human T cells induces COX-2 expression in human monocytes and synoviocytes in a autocrine manner, which is inhibited by cyclosporin A [42,43] (Figure 4). Thus, IL-17 is involved in the production of COX-2.

In addition, we recently reported that IL-17 induces human osteoclastogenesis from human monocytes even in the absence of osteoblastic cells or without adding soluble RANKL, through both inductively expressed TNF-α and constitutively expressed RANKL on human monocytes [44] (Figure 4). In this osteoclastogenesis, the synergistic effect of TNF-α and RANKL plays an important role; the expressed level of each cytokine alone is too low to induce osteoclastogenesis [44] (Figure 4). This synergism has also been reported by two other groups, Lam *et al.* in 2000 [45] and Zoo *et al.* in 2001 [46]. More recently, Miranda-Carus *et al.* reported that peripheral blood T cells from patients with early RA promote osteoclastogenesis in autologous monocytes in the absence of exogenous cytokines or osteoblasts, and that osteoclastogenesis is significantly inhibited by neutralizing monoclonal antibodies to IL-17 [47]. Thus, IL-17 induces osteoclastogenesis from monocytes both in the presence and absence of osteoblasts.

Other groups recently demonstrated that IL-17 plays a crucial role in the pathogenesis of RA. In 2005, Raza *et al.* reported that early RA of 3-month duration (mean: 9 weeks) or less is characterized by a distinct and transient synovial fluid cytokine profile of T cells, including IL-17, but not IFN-γ [48]; this study underlined the fact that the disease duration is very important in studies on the role of cytokines in the pathogenesis of diseases. In 2010, two other groups reported important findings. Kokkonen *et al.* reported that LY2439821, a humanized anti-IL-17 monoclonal antibody, added to oral DMARDs improves the signs and symptoms of RA, with no strong adverse safety signal noted [49]. This first evaluation of LY2439821 supports the neutralization of IL-17 as a potential novel target for the treatment of RA. In addition, Genovese *et al.* reported that the serum concentration of IL-17 in individuals 3.3 years pre–dating onset was significantly higher than in patients 7.7 months after disease onset [50]. Thus, these findings, in addition to the report by Raza *et al.*, mentioned above, strongly support that IL-17 clinically plays an important role in pre-onset, early phases, and chronic phases; all the phases of RA.

Through induction of the expression of IL-17, IL-23 induces human osteoclastogenesis. In 2007, we reported that IL-23 induces human osteoclastogenesis via IL-17 *in vitro*, and anti-IL-23 antibody attenuates collagen-induced arthritis in rats [51]. IL-23 induces human osteoclastogenesis in cultures of peripheral blood mononuclear cells (PBMC) in the absence of osteoblasts or exogenous soluble-RANKL. This IL-23-induced osteoclastogenesis is inhibited by OPG, anti-IL-17 antibody, and etanercept, suggesting that RANKL, IL-17, and TNFα are involved. In addition, we found that the ratio of the production levels of IL-17 to those of IFN-γ from activated human T cells is elevated at 1 to 10 ng/mL of IL-23. The inductive effect of IL-17 and the inhibitory effect of IFN-γ on osteoclastogenesis indicate that the balance of these two cytokines is particularly important. We also demonstrated that anti-IL-23 antibody administered at a later stage significantly reduces paw volume in rats with collagen-induced arthritis, in a dose-dependent manner. Furthermore, anti-IL-23 antibody reduces synovial tissue inflammation and bone destruction in these rats. These findings suggest that IL-23 is important in human osteoclastogenesis and that neutralizing IL-23 after the onset of collagen-induced arthritis has therapeutic potential. Thus, controlling IL-23 production and function could be a strategy for preventing inflammation and bone destruction in patients with rheumatoid arthritis [51].

## 6. Effects of PGE2 on Mature Osteoclasts

The actions of PGs on the functions of mature osteoclasts, but not osteoclastogenesis, have been reported. In 1984, Chambers *et al.* found that PGs acts as direct inhibitors of osteoclastic spreading using osteoclasts isolated from neonatal rat bone [52]; however, if osteoblasts and osteoclasts are co-cultured, the addition of PGs causes a considerable increase in their spread suggesting that osteoblasts possess the capacity to either inhibit through PGs or stimulate osteoclasts. In 1989, Chambers *et al.* found that PGE1 and PGE2 inhibited bone resorption by isolated rats osteoclasts for at least 6 h and that the inhibition was followed by recovery to control, not supranormal levels [53]. Thus, PGE2 inhibits the function of mature murine osteoclasts.

Osteoclasts express unique cell adhesion structures called podosomes, which contain actin filaments. Podosomes are organized differently depending on the activity of the osteoclast; in bone-resorbing osteoclasts, podosomes form the actin ring, representing a gasket-like structure, necessary for bone resorption, and in motile osteoclasts, podosomes are organized into lamellipodia (Latin *lamella*, a thin leaf; Greek *pous*, foot), the structure responsible for cell movement. Thus, the presence of actin rings and lamellipodia is mutually exclusive [54]. In 2004, Sarrazin *et al.* showed, using mature human osteoclasts extracted from the femurs and tibias of human fetuses, that osteoclasts have two subtypes of EP receptors, EP3 and EP4, that mediate different actions of PGE2 on these cells; activation of EP4 receptors inhibits actin ring formation and activation of EP3 receptors increases the number of lamellipodia [54] (Figure 3). Thus, PGE2 directly inhibits bone resorption by human osteoclasts.

In 2006, Hackett *et al.*, the same group as Sarrazin *et al.*, demonstrated that human osteoclasts express COX-1, COX-2, and cytosolic phospholipids A2 using both human osteoclasts extracted from human fetuses and human osteoclasts differentiated from peripheral blood mononuclear cells [55]. Interestingly, inhibition of COX-1 increases resorption pit formation by osteoclasts, whereas inhibition of COX-2 has no effect. These findings suggest that PGs produced mainly through the COX-1 pathway seem particularly implicated in the inhibition of human bone resorption (Figure 3). Thus, PGs play an important role in the function of mature osteoclasts as well as in osteoclastogenesis.

## 7. COX-2 Inhibitor and Nonselective NSAIDs

### 7.1. Diclofenac sodium

Dicrofenac directly inhibits the differentiation and function of mouse osteoclasts. In 1998, Yin *et al.* reported that the anti-inflammatory properties of aspirin are mediated in part by their specific inhibition of IKK-β, thereby preventing the activation of nuclear factor κB (NF-κB) of genes involved in the pathogenesis of the inflammation response [56]. In 2004, Jimi *et al.* demonstrated that selective inhibition of NF-κB blocks osteoclastogenesis and prevents inflammatory bone destruction in collagen-induced arthritis of mice [57]. In 2009, Karakawa *et al.* demonstrated that diclofenac has a direct effect on mouse osteoclast differentiation and activation, and that, in part, inhibition of phosphorylated NF-κB translocation is indispensable for diclofenac efficacy [58]. Interestingly, the diclofenac dose inhibiting osteoclast activation is lower than the dose that inhibits osteoclast differentiation; diclofenac strongly suppresses mature osteoclast activation. Thus, diclofenac sodium inhibits NF-κB transcription in mouse osteoclasts. 

### 7.2. Etodolac

Etodolac is a NSAID that inhibits both COX-1 and COX-2 and is approved for the treatment of degenerative joint disease and rheumatoid arthritis. In 2007, Feng *et al.* demonstrated that the tetrahydropyrano-indole structural analog of etodolac, SDX-308, effectively inhibits human osteoclast formation and activity, and multiple myeloma cell proliferation, potentially by controlling NF-κB activation signaling via a COX-2-independent mechanism [59]. Importantly, they used both human non-adherent mononuclear cells from bone marrow and human colony-forming units granulocyte–macrophage (CFU–GM) from CD34+ cells in bone marrow cells as osteoclast precursors in the presence of RANKL. Thus, SDX-308 is a therapeutic candidate to inhibit osteoclast activity and function in destructive bone lesions.

### 7.3. Celecoxib

Celecoxib directly inhibits the osteoclastogenesis induced by RANKL as well as indirect effects that inhibit PGE2 production from osteoblasts. In 2005, Han *et al.* reported that RANKL selectively induces COX-2 expression via Rac1, which in turn results in the production of PGE2 in mouse osteoclast precursors, RAW264.7 cells [33]. Blockade of COX-2 by celecoxib inhibits the differentiation of bone marrow-derived monocyte/macrophage precursor cells into osteoclasts. This inhibition is rescued by the addition of exogenous PGE2, suggesting that COX-2-dependent PGE2 induction by RANKL in osteoclast precursors is required for osteoclast differentiation [33]. In addition, Kawashima *et al.* reported that celecoxib directly inhibits human osteoclastogenesis from peripheral mononuclear cells induced by RANKL [60]. Thus, celecoxib directly inhibits osteoclastogenesis from osteoclast precursors of both mice and humans. 

On the other hand, it has been reported that celecoxib may exacerbate osteopenia in an inflammatory animal model. In 2007, Niki *et al.* demonstrated that the administration of celecoxib reduces joint inflammation, but exacerbates osteopenia in IL-1α transgenic mice, which is explained by the *in vitro* findings that celecoxib decreases osteogenesis by transgenic mouse-derived primary osteoblasts [61]. However, further detailed analyses are necessary in both *in vitro* studies using human cells and in patients with inflammatory disease.

### 7.4. Flurbiprofen derivative HCT1026

Flurbiprofen is a member of the phenylalkanoic acid derivative family of NSAIDs. In 2004, Idris *et al.* showed that the nitrosylated flurbiprofen derivative HCT1026 inhibited bone resorption, both *in vivo* and *in vitro*, and that its mechanism of action is independent of nitric oxide release and PG synthesis inhibition [62]. In 2009, they reported the effects of HCT1026 on osteoclast formation, activity, survival and cell signaling *in vitro*, as follows [63]. HCT1026 strongly inhibits osteoclast formation, activity and survival in murine osteoclast cultures, whereas macrophages and osteoblasts were unaffected. HCT1026 induces osteoclast apoptosis, which is partially prevented by increasing the concentration of RANKL, suggesting that HCT1026 inhibits bone resorption by inhibiting the effects of RANKL. In fact, HCT1026 inhibits RANKL-induced activation of NF-κB and extracellular signal-regulated kinase (ERK) pathways in osteoclasts. In addition, HCT1026 inhibits TNF-, IL1- and LPS-induced signaling, which shares a similar kinase complex upstream of the NF-κB and ERK pathways. Thus, HCT1026 could represent a novel class of anti-inflammatory compounds.

## 8. Effect of NSAIDs on Osteoblasts

In 2007, Kellinsalmi *et al.* reported that indomethacin, parecoxib and NS398, all tested COX-inhibitors, inhibit osteoblast differentiation from human mesenchymal stem cells; it is notable that the ultimate manifestation of osteoblast activity, mineral deposition, is diminished, while simultaneously, the number of adipocytes is significantly increased. These data suggest that COX-2 inhibition diverts human stem cell differentiation towards an adipocyte lineage instead of an osteoblast lineage, suggesting cautious use of COX-2 inhibitors after osseous trauma [64] (Figure 3).

## 9. Systemic Effects of NSAIDs on Bone Metabolism

PGE2 plays an important role in the regulation of osteoclast and osteoblast functions. Results of fracture healing studies in animals treated with NSAID or in mice lacking the COX-2 gene show that inhibition of COX-2 by non-selective or COX-2-selective NSAIDs delays fracture healing; however, as described above, PGE2 shows different effects on cells from humans and mice. In particular, it has been reported that PGE2 potently inhibit human osteoclastogenesis from monocytes stimulated by RANKL [6]. In 2005, Goodman *et al.* reported that the effects of COX-2 inhibitors on bone are less profound when the drug is administered for a short period of time using a well-defined rabbit model [65]. Thus, the clinical significance of the effect in various patient groups needs to be carefully assessed and further investigations are needed to characterize patients at the highest risk for NSAID-induced delayed fracture healing and its complications [66].

## 10. Geranylgeranylacetone Inhibits the Formation and Function of Human Osteoclasts

The anti-ulcer drug geranylgeranylacetone (GGA), known as teprenon, is frequently used with NSAIDs in Japan. In 2005, we demonstrated that GGA inhibits the formation and function of human osteoclasts and prevents bone loss in tail-suspended rats and ovariectomized rats [67]. Vitamin K is also used to protect against osteoporosis. It has been reported that the inhibitory effect of vitamin K2 (menatetrenone) on bone resorption may be related to its side chain. GGA has almost the same chemical structure as the side chain of menatetenone. We hypothesized that GGA also has an inhibitory effect on osteoclastogenesis both *in vitro* and *in vivo*. GGA in pharmacological concentrations directly inhibited osteoclastogenesis from human monocytes induced by soluble RANKL. In addition, GGA induced the degradation of actin rings in mature osteoclasts, which was reversed by adding geranylgeranylpyrophosphatase. Moreover, GGA increased the bone mineral density of the total femur, proximal metaphysis, and diaphysis of femur in ovariectomized rats. GGA also prevented bone loss induced by hindlimb unloading in tail-suspended rats. These results indicate that GGA prevents bone loss by maintaining a positive balance of bone turnover through suppression of both the formation and activity of osteoclasts. In addition, in 2009, we also reported that GGA induces cell death in fibroblast-like synoviocytes from patients with rheumatoid arthritis [68]. Thus, GGA could be used to prevent and improve osteoporosis, especially in patients with rheumatoid arthritis. 

## 11. Conclusions

Many studies have reported that NSAIDs inhibit murine osteoclastogenesis *in vitro* and murine arthritis models *in vivo*; however, several studies using human osteoclasts have shown the effects of COX inhibitors, contradicting the findings using murine cells. Thus, it is crucial to use human cells in studies on human bone metabolism.
